# Multimodal MRI-Based Classification of Trauma Survivors with and without Post-Traumatic Stress Disorder

**DOI:** 10.3389/fnins.2016.00292

**Published:** 2016-06-24

**Authors:** Qiongmin Zhang, Qizhu Wu, Hongru Zhu, Ling He, Hua Huang, Junran Zhang, Wei Zhang

**Affiliations:** ^1^Department of Medical Information Engineering, School of Electrical Engineering and Information, Sichuan UniversityChengdu, China; ^2^Monash Medical Imaging, Monash UniversityClayton, VIC, Australia; ^3^Mental Health Center, West China Hospital of Sichuan UniversityChengdu, China

**Keywords:** post-traumatic stress disorder, structural MRI, resting-state functional MRI, gray matter volume, amplitude of low-frequency fluctuations, regional homogeneity, multi-kernel based support vector machine

## Abstract

Post-traumatic stress disorder (PTSD) is a debilitating psychiatric disorder. It can be difficult to discern the symptoms of PTSD and obtain an accurate diagnosis. Different magnetic resonance imaging (MRI) modalities focus on different aspects, which may provide complementary information for PTSD discrimination. However, none of the published studies assessed the diagnostic potential of multimodal MRI in identifying individuals with and without PTSD. In the current study, we investigated whether the complementary information conveyed by multimodal MRI scans could be combined to improve PTSD classification performance. Structural and resting-state functional MRI (rs-fMRI) scans were conducted on 17 PTSD patients, 20 trauma-exposed controls without PTSD (TEC) and 20 non-traumatized healthy controls (HC). Gray matter volume (GMV), amplitude of low-frequency fluctuations (ALFF), and regional homogeneity were extracted as classification features, and in order to integrate the information of structural and functional MRI data, the extracted features were combined by a multi-kernel combination strategy. Then a support vector machine (SVM) classifier was trained to distinguish the subjects at individual level. The performance of the classifier was evaluated using the leave-one-out cross-validation (LOOCV) method. In the pairwise comparison of PTSD, TEC, and HC groups, classification accuracies obtained by the proposed approach were 2.70, 2.50, and 2.71% higher than the best single feature way, with the accuracies of 89.19, 90.00, and 67.57% for PTSD vs. HC, TEC vs. HC, and PTSD vs. TEC respectively. The proposed approach could improve PTSD identification at individual level. Additionally, it provides preliminary support to develop the multimodal MRI method as a clinical diagnostic aid.

## Introduction

Post-traumatic stress disorder (PTSD) is newly defined as a trauma- and stressor-related disorder in the fifth edition of the Diagnostic and Statistical Manual of mental disorder (DSM-V). PTSD may develop in individuals who have experienced or witnessed severe traumatic events. This kind of psychological disorder is characterized by re-experiencing, avoidance, negative cognitions and mood, and arousal (Contractor et al., 2014). Recent surveys reported that, the prevalence of PTSD among direct victims of disasters ranges between 30 and 40%; lifetime prevalence of PTSD varies from 0.3 to 6.1% in different countries (Javidi and Yadollahie, 2012), and 19% of PTSD patients will attempt suicide (Kessler et al., 1999; Foa et al., 2006). Thus it is of paramount importance for the early diagnosis and appropriate treatment of PTSD. However, there are no reliable biomarkers that can be used to identify trauma-exposed individuals with and without PTSD at present. The diagnosis of this disorder is still very reliant on the assessment of signs and symptoms, as well as a thorough psychological evaluation. Accordingly, there has been substantial interest in exploring automated and unbiased methods to assist the diagnosis of PTSD.

In recent years, studies by structural and functional magnetic resonance imaging (MRI) have yielded tremendous advances in understanding the neural mechanisms underlying PTSD. A number of structural MRI (sMRI) studies have revealed gray matter volume (GMV) or density alterations in PTSD patients, which occur in the anterior cingulate cortex (ACC) and insular cortex within the limbic-prefrontal circuit (Meng et al., 2014), ventral ACC and orbitofrontal cortex (OFC) (Sekiguchi et al., 2013), frontal and occipital lobes (Tavanti et al., 2012), limbic and paralimbic cortices (Nardo et al., 2010), and medial prefrontal cortex (Li et al., 2014). Meanwhile, resting-state functional MRI (rs-fMRI) studies have identified altered amplitude of low-frequency fluctuations (ALFF) in patients with PTSD, in many brain areas, such as the medial prefrontal cortex and ACC (Xie et al., 2013), amygdala, anterior insula and thalamus (Yan et al., 2013), medial frontal gyrus (Yin et al., 2011), visual cortex and medial ACC (Zhu et al., 2014), and OFC (Zhu et al., 2015); moreover, changes of regional homogeneity (ReHo) have been found in the inferior parietal lobule, superior frontal gyrus, middle temporal gyrus and lingual gyrus (Yin et al., 2012), amygdala, hippocampus, thalamus, medial prefrontal cortex and dorsolateral prefrontal cortex (Zhong et al., 2015). These findings by sMRI and rs-fMRI collectively indicate that, PTSD relates not only to morphological brain alterations but also to abnormalities in spontaneous brain activities.

Most of the aforementioned findings were obtained by using mass-univariate analysis approaches, and the differences were reported at group level (Davatzikos, 2004). In clinical practice, however, these group level observations were rarely beneficial to individual diagnosis. For neuroimaging to be useful in a clinical setting, we need techniques that are capable of providing predictions at the individual level. In the past several years, the application of machine learning techniques to neuroimaging data analysis has made promising improvements in brain disease classification (Orrù et al., 2012; Haller et al., 2014). In contrast to the group comparisons that are based on mass-univariate analyses, machine learning techniques allow inference at the single-subject level, and moreover, they are sensitive to subtle and spatially distributed differences in the brain which might be undetectable in group comparison. In recent years, a growing number of studies have explored the utility of machine learning methods in classifying diseases based on imaging data, and a range of psychiatric and neurological conditions have been examined, such as Alzheimer's disease (Abdulkadir et al., 2011), autism (Ecker et al., 2010), social anxiety disorder (Frick et al., 2014), depression (Gong et al., 2011), schizophrenia (Iwabuchi et al., 2013).

With regard to the technical details of applying machine learning methods, one can either use features derived from single-modality MRI data or even a single measure, or include multi-modality features. The advantage of the latter way is that different neuroimaging modalities/measures focus on different aspects, which may provide complementary information for disease diagnosis. Therefore combining multimodal features, instead of depending on one feature, is a promising direction that worth exploration to improve classification accuracy. Recent studies have successfully applied multimodal analysis on Alzheimer's disease (Fan et al., 2008; Zhang et al., 2011; Dai et al., 2012; Liu et al., 2014b), Parkinson's disease (Long et al., 2012) and sexual dimorphism (Wang et al., 2012). However, so far as we know, most of the very few studies that performed classification on PTSD only utilized single modal imaging data (Gong et al., 2014; Niehaus et al., 2014). A very recent study (Liu et al., 2015) has explored the power of multivariate approach in classifying PTSD in which features at three different levels derived from rs-fMRI data were combined, although this should be still considered as a single-modality study. In consideration of the previous findings, we would presume that, by integrating the information derived from sMRI and fMRI data properly using multi-kernel learning methods, the discriminative power for PTSD could be further improved.

In this study, we proposed a framework to identify PTSD using both sMRI and rs-fMRI scans. Specifically, GMV, ALFF, and ReHo were extracted as classification features and effectively combined by using a simple-while-effective multi-kernel strategy. Then a support vector machine (SVM) classifier was trained to do the work and unbiased estimation of the classification performance was obtained via a leave-one-out cross-validation (LOOCV) scheme. The aim of this study was to examine whether the complementary information conveyed among structural and functional features could be combined to improve the classification performance for PTSD.

## Materials and methods

### Subjects

We recruited 37 trauma-exposed individuals who had experienced the Wenchuan 8.0-magnitude earthquake, including 17 PTSD patients (5 males and 12 females with a mean age of 44.41 ± 8.44 years) and 20 trauma-exposed controls without PTSD (TEC) (9 males and 11 females with a mean age of 40.35 ± 9.43 years). To avoid treatment-elicited changes in patient mental function, only treatment-naïve (neither psychotherapy nor pharmacotherapy) PTSD patients were recruited into the study. The diagnosis of PTSD and TEC was made with the Structured Clinical Interview for DSM-IV (SCID) and the Clinician Administered PTSD Scale (CAPS). Inclusion criteria for the trauma-exposed individuals included: (1) physically experienced the earthquake; (2) personally witnessed death, serious injury or the collapse of buildings; and (3) did not suffer any physical injury. Exclusion criteria included: (1) history of neurological disorders; (2) present or past Axis-I psychiatric disorders other than PTSD; (3) drug or alcohol abuse/dependence within the 6 months prior to the study; (4) contraindications to MRI; (5) learning or developmental disorders; or (6) a family history of mental disorders. The acquisition of neuroimaging and clinical data took place 2 years after the earthquake. In addition, 20 non-traumatized healthy controls (HC) (8 males and 12 females with a mean age of 42.52 ± 7.89 years) were recruited by advertisement. Healthy controls also satisfied the mentioned exclusion criteria. All subjects were right-handed, aged between 21 and 61 years, and underwent brain scans at the Huaxi MR Research Center of the West China Hospital. This study was approved by the Medical Ethics Committee of the West China Hospital, Sichuan University, and written informed consent was obtained from each participant.

### Image acquisition

Structural and resting-state functional MRI scans were acquired using a 3T MRI system (EXCITE, General Electric, Milwaukee, USA) with an 8-channel phased array head coil. A three-dimensional spoiled gradient-recalled (SPGR) sequence was used to collect structural scans: repetition time/echo time (TR/TE) = 8.5/3.4 ms, flip angle = 12°, slice thickness/gap = 1/0 mm, field of view (FOV) = 240 × 240 mm^2^, voxel size = 0.47 × 0.47 × 1 mm^3^. The resting-state functional images were collected using a gradient-recalled echo planar imaging (EPI) sequence: TR/TE = 2000/30 ms, flip angle = 90°, slice thickness/gap = 5/0 mm, FOV = 240 × 240 mm^2^, matrix = 64 × 64, voxel size = 3.75 × 3.75 × 5 mm^3^. Each brain volume comprised of 30 axial slices and each functional run contained 200 image volumes. During data acquisition, all participants were instructed to keep their eyes closed but not fall asleep, relax their minds, and keep still as possible.

### Data preprocessing

All structural images were preprocessed using the Statistical Parametric Mapping software (SPM8, http://www.fil.ion.ucl.ac.uk/spm). After orientation correction according to the anterior commissure—posterior commissure line, images were segmented into gray matter, white matter, and cerebrospinal fluid partitions using the segment routine in SPM8. The diffeomorphic anatomical registration through exponentiated lie algebra (DARTEL) algorithm (Ashburner, 2007) was applied to gray and white matter partitions to generate a study-specific template. Then the gray matter images were warped to the study-specific template and re-sampled to an isotropic resolution of 3 mm. All the warped and re-sampled gray matter images were modulated to assess the GMV. Finally, the modulated images underwent spatial smoothing using an 8 mm full-width at half-maximum (FWHM) Gaussian kernel. Finally, a GMV map was obtained for each subject.

Resting-state functional images were preprocessed using SPM8 and the Resting-State fMRI Data Analysis Toolkit (REST, http://rest.restfmri.net). Considering the magnetization saturation effects and participants' adaptation to the environment, the first 10 volumes of each dataset were discarded. The remaining images were first corrected for within-scan acquisition time differences between slices, and further realigned to the first volume to correct for susceptibility-by-movement interaction. All subjects in this study had less than 2 mm displacement and 2° of rotation in any direction. The realigned scans were further spatially normalized to the Montreal Neurological Institute template and resliced to 3 × 3 × 3 mm^3^ in SPM8. When calculate ALFF, the normalized and resliced images were smoothed using a 4 mm FWHM Gaussian kernel. Then the ALFF, across the frequency band 0.01–0.08 Hz (Zang et al., 2007), was calculated for each voxel using the REST software. To reduce the global effects of variability across all subjects, ALFF of each voxel was divided by the global mean ALFF value for each subject. Thus, an ALFF map was obtained for each subject. The ReHo map of each subject was calculated in REST as well. For each normalized and resliced image from SPM8, cluster size was set at 27 voxels when computing ReHo value for each voxel (26 neighbors) (Zang et al., 2004). As did in ALFF, the ReHo of each voxel was also divided by the global mean ReHo value for each subject. After smoothing with a 4 mm FWHM Gaussian kernel, a ReHo map was obtained for each subject.

### Feature extraction

We obtained the GMV, ALFF, and ReHo maps for each subject. These three features provide information from different perspectives. For a given subject, GMV map gives us the morphometric information; ALFF and ReHo reflect the degree of regional activity and the degree of regional synchronization respectively. In the present study, a whole brain mask including only brain tissue voxels was applied for all subjects. Let **x**^(1)^ = [x${}_{1}^{\left(1\right)}$, x${}_{2}^{\left(1\right)}$, …, x${}_{D}^{\left(1\right)}$], **x**^(2)^ = [x${}_{1}^{\left(2\right)}$, x${}_{2}^{\left(2\right)}$, …, x${}_{D}^{\left(2\right)}$], and **x**^(3)^ = [x${}_{1}^{\left(3\right)}$, x${}_{2}^{\left(3\right)}$, …, x${}_{D}^{\left(3\right)}$] denote feature vectors that were extracted from the GMV, ALFF, and ReHo maps. *D* is the number of voxels in the brain. Hence, each voxel *i* has three representations: GMV value x${}_{i}^{\left(1\right)}$, ALFF value x${}_{i}^{\left(2\right)}$, and ReHo value x${}_{i}^{\left(3\right)}$. Since the individual feature value of vectors **x**^(1)^, **x**^(2)^, and **x**^(3)^ may exhibit significant variations in both their range and distribution, feature normalization has always performed to ensure the contribution of each feature to the final classifications is comparable (Ross et al., 2006). We used the Z-score normalization technique in this study, that is, feature values were normalized by subtracting their mean and dividing the standard deviation, to get a zero mean, and unit variance for each feature.

### Multi-kernel based SVM

In order to effectively combine different feature vectors, the multi-kernel combination strategy (Liu et al., 2014a), and SVM were used here. After feature extraction, we constructed a kernel matrix for each feature, and combined them using a weighted linear combination as follows:

(1) $$\begin{array}{l} K\left({\text{x}}_{i},\text{x}\right)=\sum_{f=1}^{3}\beta_{f}k_{f}\left({\text{x}}_{i}^{f},{\text{x}}^{f}\right) \end{array}$$

where **x**_*i*_ is the feature vector of the *i*-th training sample; **x** is the feature vector of the test sample; *k*_*f*_(**x**${}_{i}^{f}$, **x**^*f*^) is the *f* -th kernel function; and β_*f*_ ≥ 0 is the weighting factor of *f*-th kernel function with the constraint of $\sum_{f=1}^{3}$β_*f*_ = 1.

The relationship between features and the prediction is hard to interpret when a nonlinear kernel function is used (Norman et al., 2006; Pereira et al., 2009). Therefore, we trained a SVM classifier with linear kernel functions to directly extract the weight vectors. Given a labeled training set {(**x**_*i*_, *y*_*i*_)${}_{i\;=\;1}^{n}$} denotes the training sample and *y*_*i*_ϵ{−1, +1} denotes the corresponding class label. The multi-kernel based SVM solves the following optimization problem:

(2) $$\begin{array}{cc} \underset{w^{\left(f\right)},b,\xi }{\min} & \frac{1}{2}\sum_{f=1}^{3}\beta_{f}{\left\|w^{\left(f\right)}\right\|}^{2}+C\sum_{i=1}^{n}\xi_{i}\\ s.t. & y_{i}\left(\sum_{f=1}^{3}\beta_{f}\langle w^{\left(f\right)},\Phi^{\left(f\right)}(x_{i}^{\left(f\right)})\rangle +b\right)\geq 1-\xi_{i}\\ & \xi_{i}\geq 0,i=1,2,\cdots n \end{array}$$

where **w**^(^^*f*^^)^ denotes the normal vector of hyperplane; Φ^(^^*f*^^)^ is the kernel-induced mapping function; *C* trades off the empirical risk and mode complexity; and ξ_*i*_ is the slack variable.

The dual form of multi-kernel based SVM can be represented as below:

(3) $$\begin{array}{cc} \underset{\beta }{\min}\underset{\alpha }{\max} & \sum_{i=1}^{n}\alpha_{i}-\frac{1}{2}\sum_{i,j}\alpha_{i}\alpha_{j}y_{i}y_{j}\sum_{f=1}^{3}\beta_{f}k_{f}(x_{i}^{f},x^{f})\\ s.t. & \sum_{i=1}^{n}\alpha_{i}y_{i}=0,0\leq \alpha_{i}\leq C,i=1,2,\cdots n\\ & \sum_{f=1}^{3}\beta_{f}=1,\beta_{f}\geq 0 \end{array}$$

where α_*i*_, α_*j*_ are Lagrange multipliers.

**Figure 1:**
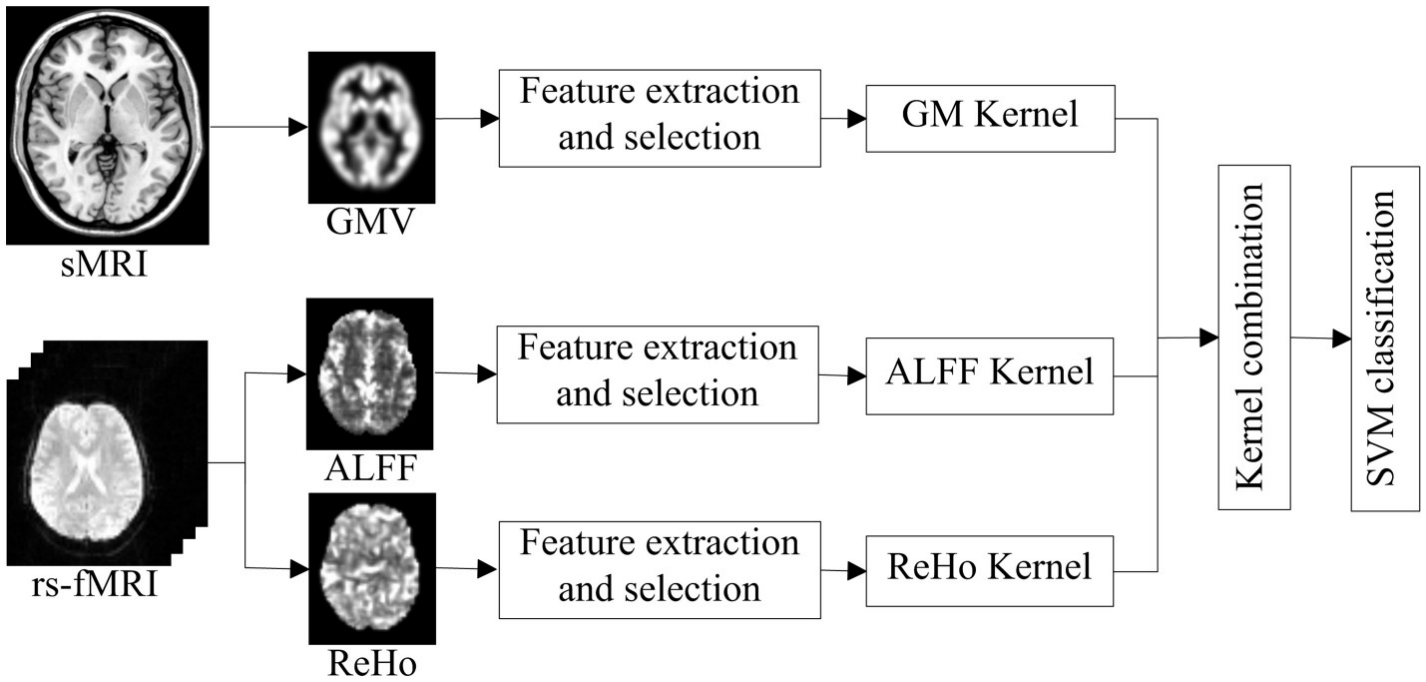
**Schematic illustration of multimodal feature combination and classification**. GMV, ALFF, and ReHo measures are used to map brain structure and resting-state function, respectively. A SVM classifier is then designed using a multi-kernel combination strategy to classify PTSD, TEC, and HC.

In this study, the algorithm was developed using the SimpleMKL Toolbox (http://asi.insa-rouen.fr/enseignants/~arakoto/code/mklindex.html). The weights of different kernels in the multi-kernel based SVM are learned based on the training samples (Rakotomamonjy et al., 2008). The optimization of kernel weights and SVM classifier are alternate: given the current solution of kernel weights, it solves a classical SVM with the combined kernel; then updates the kernel weights. This two-step process is repeated until a convergence criterion is met (Xu et al., 2010). As explained above, the multi-kernel based SVM can provide a convenient and effective way for fusing various features from different modalities. In our case, we focused on multimodal classification using two modalities: sMRI and rs-fMRI. Figure 1 gives a schematic illustration of our multimodal feature combination and classification approach.

### Cross-validation

Cross-validation is often used to assess the generalizability of a model and to ensure that the model does not overfit data. Here, we used the LOOCV strategy to validate the performance of our proposed approach. Specifically, each sample was designated as a test sample, while the remaining samples were used to train the classifier. By repeatedly repartitioning data in this way, it is possible to derive an approximately unbiased estimator of the true generalization error of the model. Optimal kernel weights and optimal SVM model were obtained in the inner cross-validation before applying it to the test set. The whole process was repeated until all samples have been left out for test. The final accuracy was computed by averaging the accuracies from all experiments. Accuracy, sensitivity and specificity are defined based on the prediction results of LOOCV, to quantify the performance of all compared methods.

(4) $$\begin{array}{r} \text{Sensitivity}=\frac{TP}{TP+FN} \end{array}$$

(5) $$\begin{array}{r} \text{Specificity}=\frac{TN}{TN+FP} \end{array}$$

(6) $$\begin{array}{r} \text{Accuracy}=\frac{TP+TN}{TP+FN+TN+FP} \end{array}$$

where *TP* denotes the number of patients correctly classified; *FN* denotes the number of patients classified as controls; *TN* denotes the number of controls that are correctly predicted; and *FP* denotes the number of controls classified as patients. In addition, the receiver operating characteristic (ROC) curve is plotted and the area under ROC (AUC) curve is calculated to illustrate the performance of classification.

### Discrimination maps

Since the input space is voxel space (one dimension per voxel) in this study, each voxel carry a certain weight value signifying its contribution toward the classification function. The larger the absolute magnitude of a weight vector is, the stronger it affects the final discrimination. Hence, a map of the most discriminating regions (i.e., a discrimination map) could be generated. Because the SVM classifier is of multivariate and the discrimination is based on the whole brain pattern (i.e., all voxels contribute to the classification), local inferences should never be made in regards to the weights. For each discrimination result, by setting the threshold to 30% of the maximum (absolute) weight value (Mourao-Miranda et al., 2012), we obtained a spatial representation of the regions that contribute most to the group discrimination.

## Results

### Demographics and clinical scores

Data from 17 earthquake survivors with PTSD, 20 trauma-exposed non-PTSD and 20 non-traumatized healthy controls was utilized in the current study. Two-sample *t*-tests were performed to assess the differences in age, years of education and clinical score, and Chi square test was performed to assess the difference in gender. There were no significant differences in terms of gender, age or years of education (*p* > 0.05) in pairwise comparison of the three groups. Compared with TEC, patients with PTSD have significant higher CAPS total score (*p* < 0.001). The detailed demographic and clinical data are shown in Table 1.

**Table 1:** **Demographic and clinical characteristics of participants**.

**Variables (mean ± SD)**	**PTSD**	**TEC**	**HC**	***p*****-value**		
				**PTSD vs. HC**	**TEC vs. HC**	**PTSD vs. TEC**
Gender (f/m)	17 (12/5)	20 (11/9)	20 (12/8)	0.50	0.75	0.33
Age (yrs)	44.41 ± 8.44	40.35 ± 9.43	42.52 ± 7.89	0.49	0.44	0.18
Education (yrs)	7.59 ± 2.50	8.90 ± 2.56	8.40 ± 2.50	0.33	0.54	0.13
CAPS (total)	59.76 ± 6.35	14.35 ± 3.77	−	−	−	<0.001

SD, standard deviation; PTSD, post-traumatic stress disorder; TEC, trauma-exposed controls without PTSD; HC, healthy controls; CAPS, Clinician Administered PTSD Scale.

### Comparison of classification performance

**Figure 2:**
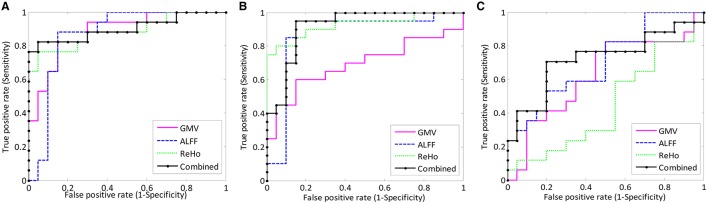
**ROC curves of different methods show the trade-off between sensitivity (y-axis) and specificity (x-axis, 1-specificity): (A) PTSD vs. HC, (B) TEC vs. HC, and (C) PTSD vs. TEC classifications**.

To examine whether or not GMV, ALFF, and ReHo features are suitable for PTSD, TEC, and HC classification, and to examine whether integrating structural and functional information could improve the classification performance, we applied single-kernel SVM classifier and multi-kernel based SVM classifier for single feature and multi-feature classification respectively. The linear SVM has only one parameter *C* that controls the trade-off between having zero training errors and allowing misclassifications. We fixed *C* = 100 for all cases. It has been shown previously that the SVM performance for whole-brain classification does not change for a large range of *C* values and only degrades with very small *C* values (LaConte et al., 2005). In the current study, LOOCV approach was used to evaluate the generalizability of different feature type classifications. Same training and test data were used in all the classifications for fair comparison. Table 2 lists the classification results of the single feature method and our multimodal feature combination method. Besides, the corresponding ROC curves were plotted (see Figure 2). The larger area under ROC is obtained, the better classification performance is achieved.

**Table 2:** **Classification performance of the single feature method and multimodal feature combined method**.

**Feature types**	**PTSD vs. HC**				**TEC vs. HC**				**PTSD vs. TEC**			
	**SEN (%)**	**SPE (%)**	**ACC (%)**	**AUC value**	**SEN (%)**	**SPE (%)**	**ACC (%)**	**AUC value**	**SEN (%)**	**SPE (%)**	**ACC (%)**	**AUC value**
GMV	64.71	85.00	75.68	0.88	60.00	85.00	72.50	0.69	64.71	55.00	59.46	0.62
ALFF	88.24	80.00	83.78	0.86	90.00	85.00	87.50	0.87	52.94	75.00	64.86	0.70
ReHo	76.47	95.00	86.49	0.89	75.00	100.00	87.50	0.93	29.41	50.00	40.54	0.44
Combined	76.47	100.00	89.19	0.90	95.00	85.00	90.00	0.92	52.94	80.00	67.57	0.72

SEN, sensitivity; SPE, specificity; ACC, accuracy; AUC, area under receiver operating characteristic curve.

In the identification of PTSD and HC, the best classification accuracy was obtained using multimodal feature combined method. Here, 89.19% of individuals were correctly assigned to the appropriate diagnostic category. The sensitivity was 76.47%, implying that 76.47% of the PTSD patients were correctly classified. The specificity was 100%, indicating that all the control subjects were correctly predicted. However, for the single feature method, the best accuracy achieved was only 86.49% when using ReHo as the feature. The ROC curves of the four feature type methods were shown in Figure 2A.

To classify TEC from HC, we yielded a similar result. Multimodal feature combined method achieved a classification accuracy of 90.00%, with a sensitivity of 95.00%, and a specificity of 85.00%. Nevertheless, the best accuracy of single feature classification was only 87.50% (when using ALFF or ReHo). ROC curves of the four feature type methods were shown in Figure 2B.

Contrasted with the two classifications above, PTSD and TEC classification performed at lower accuracy. Multimodal feature combined approach resulted into an accuracy of 67.57%, with a sensitivity of 52.94%, and a specificity of 80.00%, yet still better than the single feature classification of which the best accuracy was 64.86% (when using ALFF). ROC curves of the four feature type methods were shown in Figure 2C.

Furthermore, the accuracy scores and the ROC curves analysis (Figure 2) all showed that, in the single-feature mode, the most powerful feature is different for the three pairwise group classifications. For instance, ReHo is the best for PTSD vs. HC and TEC vs. HC, but ALFF performed best in PTSD vs. TEC. In any case, as seen in Table 2, the feature combination approach always further improved the classification accuracy in comparison with the single feature method.

### The most discriminative regions

**Figure 3:**
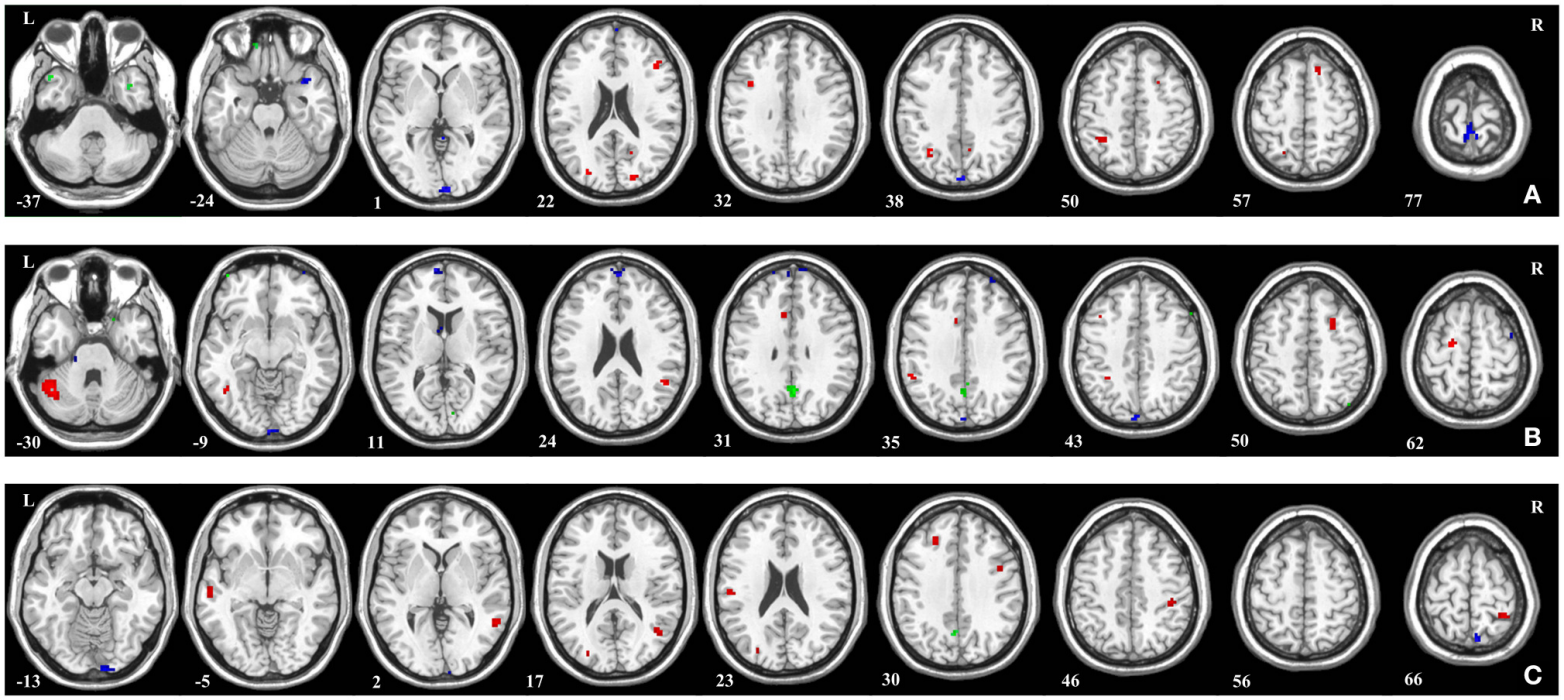
**Brain regions that showed the highest discriminative value for the classification in (A) PTSD and HC, (B) TEC and HC, and (C) PTSD and TEC**. Regions were identified by setting the threshold to 30% of the maximum (absolute) weight value. Red, blue, and green colors indicate the most discriminative regions of GMV, ALFF, and ReHo features, respectively.

Brain regions with the most discriminative power between groups were identified in both brain hemisphere and all the four lobes (Figure 3), which mean there were widespread regional alterations across the whole brain in PTSD patients as well as TEC subjects.

In comparison of PTSD and HC, regions displaying most difference in GMV appeared in the bilateral middle occipital gyrus, right inferior parietal lobule, left superior frontal gyrus, right cerebellum, and the bilateral middle frontal gyrus; ALFF difference mainly exhibited in the right precuneus, left temporal pole (superior temporal gyrus), left calcarine fissure, right caudate nucleus, and the left superior frontal gyrus (medial); ReHo difference appeared in the right temporal pole (middle temporal gyrus) (Figure 3A; see Supplementary Table 1 for a full list).

To classify TEC from HC, most discriminative regions in GMV were observed in the right inferior temporal gyrus, bilateral superior frontal gyrus, right cerebellum, left angular gyrus, right supramarginal gyrus, and the right median cingulate gyrus; ALFF difference primarily shown in the right temporal pole (superior temporal gyrus), bilateral cerebellum, left calcarine fissure, right middle frontal gyrus, left caudate nucleus, bilateral superior frontal gyrus (medial), and the right superior occipital gyrus; ReHo difference was shown in the left precuneus gyrus (Figure 3B; see Supplementary Table 2 for a full list).

In the comparison of PTSD and TEC, GMV difference mainly included the bilateral middle temporal gyrus, right rolandic operculum, right superior frontal gyrus, and the left postcentral gyrus; ALFF difference were shown in the left lingual gyrus and left precuneus gyrus; ReHo difference mainly exhibited in the right precuneus gyrus (Figure 3C; see Supplementary Table 3 for a full list).

## Discussion

To our best knowledge, this is the first study to examine the capability of a machine learning approach to combine different features extracted from sMRI and rs-fMRI data for PTSD, TEC, and HC discrimination. The results showed that in comparison with single feature method, the feature-combining framework could achieve higher accuracies for all three pairwise classifications among the three groups. This validated the efficacy of our approach in integrating effective information from multi-modality imaging data to improve classification performance.

In our single feature classifications of PTSD/TEC vs. HC, higher accuracy was obtained when using ALFF or ReHo feature than GMV feature. This may imply that in traumatized subjects the spontaneous brain activity altered in a larger extent than morphometric brain changes. In the study by Gong et al. (2014), high classification accuracy (up to 91%) has been achieved using a large sMRI dataset (50 vs. 50 vs. 40 subjects), while in a recent rs-fMRI study (Liu et al., 2015), an accuracy at 92.5% was also obtained using only 20 subjects per group. Based on this, we would cautiously conclude that fMRI features could contribute more than sMRI in PTSD identification. But in the comparison of PTSD and TEC, we noticed that the classification accuracies of all three features were lower, especially ReHo (40.54%) was much lower than ALFF (64.86%) and GMV (59.46%). Perhaps the difference between PTSD and TEC in the same MRI modality was feature-specific; yet another possibility is that PTSD and TEC subjects all experienced traumatic events so their imaging marker do not differ as much as when comparing to HC group.

In multi-feature classification, the feature combining method is an important technical point, especially when dealing with multimodality imaging data. A common and simple practice is to concatenate all features into a longer feature vector, however this may not be enough to ensure effective information integration. In this study, we combined features from different modalities using a multi-kernel combination strategy, which firstly combined the kernel matrices of different features into a mixed kernel matrix, and from which to train a single SVM model. Compared to the direct concatenation method, multi-kernel combination strategy offered more flexibility of assigning different kernel weights to different features. The improved classification performances verified the superiority of this combination strategy.

The most discriminative regions we identified by the proposed approach were widespread and not restrict to particular brain hemispheres or lobes. In SVM techniques, there are two possible reasons that an individual region could display high discriminative power: (1) a feature value difference between groups in that region; and (2) a difference in the correlation between that region and other areas between groups. Thus, the widespread network revealed in this kind of studies should not be interpreted as individual regions but a spatially distributed pattern, and the discrimination was informed by all voxels in the brain. So it's difficult to directly compare our results with previous reported sMRI and rs-fMRI studies that employed mass-univariate analyses. However, intuitively one would assume that brain regions showing great difference in group comparison should also contribute more to the SVM based classification. By a brief review we found that the discriminative regions revealed here by setting a 30% threshold were partially overlapped with previous PTSD studies. For example, volumetric MRI studies have reported that relative to HC, PTSD patients presented significant gray matter density changes in the middle frontal gyrus and inferior parietal lobule (Sui et al., 2010), GMV reductions in the frontal and occipital lobes (Tavanti et al., 2012); compared with TEC, PTSD patients showed decreased GMV in the temporal gyrus (Kühn and Gallinat, 2013) and prefrontal cortex (Nardo et al., 2013); in rs-fMRI studies, decreased ALFF values in the precuneus gyrus (Yan et al., 2013) and lingual gyrus (Yin et al., 2011) of PTSD subjects have been reported. Particularly, several limbic regions have been identified with high discrimination power, this is consistent with the recent observations that structural alterations or dysfunctions of the limbic regions are closely associated with PTSD (Lanius et al., 2010; Nardo et al., 2010). Even so, our discriminative regions did not completely replicate those sMRI or rs-fMRI findings.

Going through the very few PTSD classification studies, we found the one used structural imaging feature (Gong et al., 2014) also identified a widely distributed brain network that comprised all brain lobes. In the other two studies that used task fMRI (Niehaus et al., 2014) and rs-fMRI (Liu et al., 2015) data respectively, limbic and prefrontal areas were all considered played key role in discriminating PTSD subjects with healthy controls that showed certain consistency with our discrimination maps. It is also worth noting that the network pattern depends on the threshold selection, so the regions shown on discrimination maps only indicate their relatively high contributions.

Several limitations of this study should be noted. Firstly, we only included sMRI and rs-fMRI data into the multimodal classification, which is truly a “bimodal,” though three features were used. We attempted to acquire data of extra modalities (event-related fMRI, diffusion MRI and electroencephalogram etc.), but the number of subjects was not yet enough for a reasonable classification. Extra modalities data collection and utilization will be considered in the further. Secondly, the PTSD, TEC, and HC groups were compared only in a pairwise way. Multi-class classification approach is a direction to explore to see whether the accuracy could be further improved. Finally, given that a small sample (57 subjects in total) was used in this study, the obtained classifier is somewhat cohort specific. Next, we would use a larger dataset to determine the generalizability of the proposed approach.

## Conclusion

This study proposed a novel framework to discriminate PTSD, TEC, and HC using features derived from sMRI and rs-fMRI scans. Single feature classification results revealed that GMV, ALFF, and ReHo features could be used to identify PTSD/TEC at individual level. Compared with the single feature method, improved classification performance was obtained by combining multimodal features via multi-kernel based SVM. The promising classification results provide preliminary support to develop this multimodal MRI approach toward assisting clinical practice, which can potentially improve the clinical diagnosis of PTSD, as well as other brain disorders.

## Author contributions

QZ, QW, HZ: data analysis, interpretation of data for the work, drafting the manuscript, final approval of the version to be published and agreement to be accountable for all aspects of the work. LH, HH: interpretation of data for the work, revising the manuscript, final approval of the version to be published, and agreement to be accountable for all aspects of the work. JZ: conception and design the work, revising the manuscript, final approval of the version to be published, and agreement to be accountable for all aspects of the work. WZ: data acquisition, revising the manuscript, final approval of the version to be published, and agreement to be accountable for all aspects of the work.

### Conflict of interest statement

The authors declare that the research was conducted in the absence of any commercial or financial relationships that could be construed as a potential conflict of interest.
