# Supplementary material for: Multimodal MRI-Based Classification of Trauma Survivors with and without Post-Traumatic Stress Disorder
Source: Front Neurosci. 2016 Jun 24;10:292. doi: 10.3389/fnins.2016.00292 (PMC4919361; doi:10.3389/fnins.2016.00292)
Supplement: Supplementary file 1 [file Table1.DOC]

Supplementary Material

# Multimodal MRI-Based Classification of Trauma Survivors with and without Post-traumatic Stress Disorder

Qiongmin Zhang^1†^, Qizhu Wu^2†^, Hongru Zhu^3†^, Ling He^1^, Hua Huang^1^, Junran Zhang^1*^, Wei Zhang^3*^

*** Correspondence:**

Junran Zhang: [zhangjunran@126.com](mailto:zhangjunran@126.com)

Wei Zhang: [weizhang27@163.com](mailto:weizhang27@163.com)

**Supplementary Table 1. The most discriminating regions revealed by the GMV, ALFF and ReHo discriminative map** **(in the top 30% of the maximum absolute weight vector score), for the comparison between PTSD and HC.** The *w_i_* refers to the peak weight vector score in each cluster. PTSD, post-traumatic stress disorder; HC, non-traumatized healthy controls.

| **Features** | **Regions** | **Cluster size** | **MNI Coordinate (x, y, z)** | ***w_i_* (×10^-2^)** |
| --- | --- | --- | --- | --- |
| **GMV** | ***PTSD*>*HC*** | | | |
|  | Right middle occipital gyrus | 13 | -33, -60, 39 | 1.95 |
|  | Right inferior parietal lobule | 19 | -33, -48, 51 | 1.66 |
|  | Left superior frontal gyrus | 3 | 15, 39, 45 | 1.52 |
|  |  | 9 | 15, 24, 57 | 1.64 |
|  | ***PTSD*<*HC*** | | | |
|  | Right cerebellum | 5 | -27, -54, -57 | -1.49 |
|  | Left middle occipital gyrus | 9 | 21, -84, 21 | -1.80 |
|  | Right middle occipital gyrus | 9 | -27, -81, 18 | -1.75 |
|  | Left middle frontal gyrus | 12 | 45, 30, 21 | -1.70 |
|  | Right middle frontal gyrus | 10 | -36, 12, 33 | -1.67 |
| **ALFF** | ***PTSD*>*HC*** | | | |
|  | Right caudate nucleus | 3 | -6, 3, 9 | 2.20 |
|  | Left superior frontal gyrus, medial | 3 | 3, 66, 24 | 2.30 |
|  | ***PTSD*<*HC*** | | | |
|  | Left temporal pole: superior temporal gyrus | 6 | 39, 15 -24 | -3.02 |
|  | Left calcarine fissure | 4 | 6 -99, 3 | -2.65 |
|  | Left cuneus | 9 | 0, -87, 39 | -2.95 |
|  | Right precuneus | 13 | 0, -39, 78 | -3.05 |
| **ReHo** | ***PTSD*>*HC*** | -- | -- | -- |
|  | ***PTSD*<*HC*** | | | |
|  | Right temporal pole: middle temporal gyrus | 3 | -42, 18 -36 | -3.87 |
