# Supplementary material for: Multimodal MRI-Based Classification of Trauma Survivors with and without Post-Traumatic Stress Disorder
Source: Front Neurosci. 2016 Jun 24;10:292. doi: 10.3389/fnins.2016.00292 (PMC4919361; doi:10.3389/fnins.2016.00292)
Supplement: Supplementary file 2 [file Table2.DOC]

Supplementary Material

# Multimodal MRI-Based Classification of Trauma Survivors with and without Post-traumatic Stress Disorder

Qiongmin Zhang^1†^, Qizhu Wu^2†^, Hongru Zhu^3†^, Ling He^1^, Hua Huang^1^, Junran Zhang^1*^, Wei Zhang^3*^

*** Correspondence:**

Junran Zhang: [zhangjunran@126.com](mailto:zhangjunran@126.com)

Wei Zhang: [weizhang27@163.com](mailto:weizhang27@163.com)

**Supplementary Table 2. The most discriminating regions revealed by the GMV, ALFF and ReHo discriminative map** **(in the top 30% of the maximum absolute weight vector score), for the comparison between TEC and HC.** The *w_i_* refers to the peak weight vector score in each cluster. TEC, trauma-exposed controls without PTSD; HC, non-traumatized healthy controls.

| **Features** | **Regions** | **Cluster size** | **MNI Coordinate (x, y, z)** | ***w_i_* (×10^-2^)** |
| --- | --- | --- | --- | --- |
| **GMV** | ***TEC*>*HC*** | | | |
|  | Right inferior temporal gyrus | 6 | -42 -57, -9 | 1.70 |
|  |  | 3 | -45, -69, -3 | 1.48 |
|  | Right inferior parietal lobule | 3 | -33, -45, 42 | 1.56 |
|  | Right superior frontal gyrus | 16 | -18, -9, 63 | 1.83 |
|  | ***TEC*<*HC*** | | | |
|  | Right cerebellum | 3 | -27, -54, -57 | -1.45 |
|  |  | 68 | -42, -54, -30 | -1.81 |
|  | Right middle temporal gyrus | 6 | -63, -33, -12 | -1.56 |
|  | Left superior frontal gyrus | 8 | 24, 51, 15 | -1.59 |
|  | Right middle occipital gyrus | 3 | -27, -81, 18 | -1.49 |
|  | Right inferior frontal gyrus, triangular part | 5 | -42, 30, 18 | -1.50 |
|  | Left angular gyrus | 10 | 48, -51, 27 | -1.76 |
|  | Right median cingulate gyrus | 10 | -9, 15, 33 | -1.54 |
|  | Right supramarginal gyrus | 11 | -54, -42, 36 | -1.75 |
|  | Left middle frontal gyrus | 15 | 24, 15, 48 | -1.68 |
| **ALFF** | ***TEC*>*HC*** | | | |
|  | Right temporal pole: superior temporal gyrus | 17 | -45, 9, -24 | 1.85 |
|  | Right cerebellum | 15 | -12, -30, -21 | 1.84 |
|  | Left cerebellum | 8 | 57, -63, -27 | 1.65 |
|  |  | 3 | 12, -33, -15 | 1.47 |
|  | Left calcarine fissure | 38 | 9, -99, -12 | 2.32 |
|  | Right lingual gyrus | 5 | -27, -90, -18 | 1.76 |
|  |  | 4 | -24, -96, -15 | 1.36 |
|  | Right middle frontal gyrus | 10 | -33, 60, 0 | 1.91 |
|  | Left caudate nucleus | 12 | 6, 9, 3 | 1.86 |
|  | Right superior temporal gyrus | 3 | -60, 0, 3 | 1.43 |
|  | Left superior frontal gyrus | 6 | 21, 69, 6 | 1.47 |
|  |  | 4 | 30, 0, 69 | 1.52 |
|  | Right superior frontal gyrus, medial | 11 | -3, 60, 9 | 1.65 |
|  | Left superior frontal gyrus, medial | 13 | 3, 63, 21 | 1.43 |
|  |  | 3 | 12, 66, 30 | 1.61 |
|  | Left middle frontal gyrus | 5 | 27, 57, 33 | 1.74 |
|  |  | 6 | 45, 0, 60 | 1.70 |
|  | Left precentral gyrus | 4 | 33, -27, 72 | 1.40 |
|  | Right supplementary motor area | 3 | -6, 12, 72 | 1.50 |
|  | Left supplementary motor area | 5 | 9, -3, 78 | 1.78 |
|  | ***TEC*<*HC*** | | | |
|  | Left temporal pole: superior temporal gyrus | 3 | 39, 15, -24 | -2.79 |
|  | Right superior occipital gyrus | 10 | -3, -87, 42 | -3.12 |
| **ReHo** | ***TEC*>*HC*** | | | |
|  | Left precuneus gyrus | 33 | 3, -57, 30 | 3.87 |
|  | ***TEC*<*HC*** | | | |
|  | Right temporal pole: middle temporal gyrus | 9 | -30, 18, -33 | -3.89 |
|  |  | 3 | -42, 18, -36 | -3.35 |
