# Supplementary material for: Multimodal MRI-Based Classification of Trauma Survivors with and without Post-Traumatic Stress Disorder
Source: Front Neurosci. 2016 Jun 24;10:292. doi: 10.3389/fnins.2016.00292 (PMC4919361; doi:10.3389/fnins.2016.00292)
Supplement: Supplementary file 3 [file Table3.DOC]

Supplementary Material

# Multimodal MRI-Based Classification of Trauma Survivors with and without Post-traumatic Stress Disorder

Qiongmin Zhang^1†^, Qizhu Wu^2†^, Hongru Zhu^3†^, Ling He^1^, Hua Huang^1^, Junran Zhang^1*^, Wei Zhang^3*^

*** Correspondence:**

Junran Zhang: [zhangjunran@126.com](mailto:zhangjunran@126.com)

Wei Zhang: [weizhang27@163.com](mailto:weizhang27@163.com)

**Supplementary Table 3. The most discriminating regions revealed by the GMV, ALFF and ReHo discriminative map (in the top 30% of the maximum absolute weight vector score), for the comparison between PTSD and TEC.** The *w_i_* refers to the peak weight vector score in each cluster. PTSD, post-traumatic stress disorder; TEC, trauma-exposed controls without PTSD.

| **Features** | **Regions** | **Cluster size** | **MNI Coordinate (x, y, z)** | ***w_i_* (×10^-2^)** |
| --- | --- | --- | --- | --- |
| **GMV** | ***PTSD*>*TEC*** | | | |
|  | Right middle occipital gyrus | 3 | -30, -60, 39 | 2.51 |
|  | ***PTSD*< *TEC*** | | | |
|  | Right cerebellum | 3 | -15, -45, -57 | -1.33 |
|  | Right middle temporal gyrus | 18 | -57, -18, -6 | -1.51 |
|  | Left middle temporal gyrus | 37 | 54, -51, 6 | -1.82 |
|  |  | 11 | 45, -60, 18 | -1.58 |
|  | Right middle occipital gyrus | 4 | -24, -93, 6 | -1.43 |
|  | Left middle frontal gyrus | 3 | 27, 51, 12 | -1.34 |
|  | Right superior occipital gyrus | 7 | -27, -81, 21 | -1.58 |
|  | Right rolandic operculum | 11 | -54, -21, 24 | -1.61 |
|  | Left precuneus | 3 | 18, -63, 27 | -1.36 |
|  | Right angular gyrus | 4 | -45, -57, 33 | -1.49 |
|  | Right superior frontal gyrus | 15 | -21, 36, 33 | -1.65 |
|  | Left middle frontal gyrus | 6 | 24, 39, 30 | -1.53 |
|  | Left precentral gyrus | 8 | 42, 6, 36 | -1.37 |
|  | Right precentral gyrus | 3 | -39, -3, 48 | -1.37 |
|  | Left postcentral gyrus | 18 | 39, -33, 48 | -1.59 |
|  |  | 18 | 36, -45, 66 | -1.60 |
| **ALFF** | ***PTSD*>*TEC*** | -- | -- | -- |
|  | ***PTSD*<*TEC*** | | | |
|  | Left lingual gyrus | 16 | 12, -99, -12 | -3.32 |
|  | Left precuneus gyrus | 6 | 6, -66, 66 | -2.92 |
| **ReHo** | ***PTSD*>*TEC*** | -- | -- | -- |
|  | ***PTSD*<*TEC*** | | | |
|  | Right precuneus gyrus | 6 | -3, -63, 36 | -3.33 |
